# Optimal Development of Youth Athletes Toward Elite Athletic Performance: How to Coach Their Motivation, Plan Exercise Training, and Pace the Race

**DOI:** 10.3389/fspor.2019.00014

**Published:** 2019-08-20

**Authors:** Stein G. P. Menting, David T. Hendry, Lieke Schiphof-Godart, Marije T. Elferink-Gemser, Florentina J. Hettinga

**Affiliations:** ^1^Department of Sport, Exercise and Rehabilitation, Faculty of Health and Life Sciences, Northumbria University, Newcastle upon Tyne, United Kingdom; ^2^Center for Human Movement Sciences, University Medical Center Groningen, University of Groningen, Groningen, Netherlands

**Keywords:** pacing, performance, youth, motivation, monitoring, training

## Abstract

Elite athletes have invested many years in training and competition to reach the elite level. One very important factor on the road to elite performance is the decision-making process regarding the regulation of effort over time, termed as pacing behavior. The regulation of effort is vital for optimal athletic performance during a single race and over a longer period of time (e.g., a competitive season) as an inadequate regulation could result in a higher risk of injuries, overtraining, and drop-out. Despite this, there is limited knowledge on how young athletes learn and develop the abilities related to pacing. Pacing behavior of athletes develops from childhood throughout adolescence and is thought to be closely connected to physical maturation, the development of pre-frontal cortical related (meta-) cognitive functions, as well as the gathering of experience with exercise tasks. The motivation of an athlete can critically influence how an athlete paces a single race, but also how they distribute their effort over a longer period of time. Coaches are advised to closely monitor the development of pacing behavior during adolescence (e.g., by gathering split times, and related physiological measurement, during training and competition), as well as the underlying factors including physical maturation (meta-) cognitive development and the motivation of young athletes. Furthermore, pacing behavior development could be aided by providing training in which the task, individual, and environment are manipulated. Hereby, presenting athletes with the opportunity to gain experience in situations which closely resemble the perceptual-motor conditions of upcoming competitions.

## Introduction

The athletes performing at the 2019 IAAF world championships represent the elite of the athletics community. Despite there being multiple pathways toward achieving elite athletic performance in adulthood, the dedication of these athletes might be best exemplified through the amount of time spent on training and improvement (Ericsson et al., 2018). The longevity of this dedication is vast, with published reports indicating that elite athletes may commence participating in athletics from childhood (6–7 years old), yet the mean age of peak performance of these elite athletes ranges from 24.5 to 29 (Haugen et al., 2018). Through these many years of development, a multitude of skills are to be acquired and refined by expert athletes. Failure to acquire or refine certain skills could be the reason why some junior champions fail to win as adults. One of those skills, relevant to current and future athletic champions, currently experiences a vast increase in attention from the academic world: pacing.

Pacing is defined as the goal-directed distribution of energy over a pre-determined exercise task (Edwards and Polman, 2013), a process of decision-making regarding how and when to expend energy (Smits et al., 2014). The outcome of this process is termed the pacing behavior of the athlete (Smits et al., 2014). Previous literature concerning pacing has generally focussed on analyzing the effects of the energy distribution of athletic performance. In athletic disciplines in which the performance time is under 30 s (e.g., 100 and 200 m sprints), the most beneficial distribution of energy seems to be an all-out burst (Abbiss and Laursen, 2008). During an 800 m event, elite adult athletes exhibit a pacing behavior in which the velocity decreases over the race (Hettinga et al., 2019). During the longer events (1,500 m, 5-km, 10-km and the marathon), elite adult athletes exhibit a pacing behavior in which the velocity increases over the race (Hettinga et al., 2019). Additionally, in the longer the event, there seems to be a greater requirement to more evenly distribute energy resources over the race, as the race distance increases (Hettinga et al., 2019). The pacing behavior of athletes is influenced by the specific nature of the task (Hettinga et al., 2009; Stoter et al., 2016), factors associated with the individual athlete (Micklewright et al., 2010; Williams et al., 2014), and the environment (Hettinga et al., 2017; Konings and Hettinga, 2018). While the majority of research regarding pacing behavior focuses on the relation between pacing behavior and performance during a single race or event, adequate energy distribution over longer time periods should also be considered (Edwards and Noakes, 2009). For example, the distribution of effort over a training session, tournament, season or even a 4 year Olympic cycle, could influence (current and future) performance as well as injury and drop-out rate (Schiphof-Godart and Hettinga, 2017). The long term distribution of effort is suggested to be related to the motivation and drive of the athlete (Schiphof-Godart and Hettinga, 2017). Although an athlete's drive can function as a strong motivator, it can also push athletes toward a less than ideal distribution of energy. It is thus of crucial importance to guide and coach athletes toward developing adequate pacing behavior, which will allow them to perform to the best of their ability while staying healthy, engaged and injury-free.

Although the pacing behavior of elite senior athletes has been studied extensively in the last 30 years, there is limited understanding of the processes underpinning the acquisition of pacing skills in developing junior athletes (Elferink-Gemser and Hettinga, 2017). This is remarkable, as it has previously been put forward that the skill to adequately distribute energy to achieve an exercise goal is not fully innate, but develops relative to the (meta-) cognitive and physical attributes of an individual (Micklewright et al., 2012) as well as the amount of experience an individual has with an exercise task (Foster et al., 2009). Adolescence is a period in which there are changes in an athletes physical attributes and (meta-) cognition (Beunen et al., 1992; Blakemore et al., 2010). Additionally, during the adolescent time period, many athlete programs increase the amount of training and competition, providing athletes with more opportunities to gather experience with an exercise task. Indeed, the small number of studies which have investigated the development of pacing behavior in junior athletes see a development of behavior during adolescence (Wiersma et al., 2017; Menting et al., 2019b). Despite these findings, previous research has focussed more on how the pacing behavior of elite senior athletes resulted in optimal performance, and much less so on the process of how junior athletes acquire and develop the skills to optimally pace a race (Hettinga, 2018). With pacing behavior being vital to successful performance (Konings and Hettinga, 2018), it is important to develop our understanding of how the complex skills associated with adequate energy distribution are developed across the developmental continuum. Therefore, this perspective aimed to investigate the development of pacing behavior of young athletes, specifically during adolescence. The current perspective does not only describe how does pacing behavior develop in young athletes, but will also answer additional related questions, including: what are the underlying mechanisms of pacing behavior development during adolescence? How does the motivation and drive of the athlete impact the distribution of effort in the long term? And lastly, how can coaches monitor and train pacing behavior development in elite athletes of the future?

## The Development Pathway of Pacing Behavior

A logical first step would be to map the process of pacing behavior development in athletes. It should be pointed out that although pacing behavior is influenced by the characteristics of the performed exercise task, a more general pacing skillset is present in all athletes, regardless of the specific sport they participate in (Elferink-Gemser and Hettinga, 2017). Therefore, in discussing the development of pacing behavior in athletics, we should not exclusively take into account research that focuses on athletic disciplines, but also on other sports and particularly studies exploring younger individuals (Table 1).

**Table 1:** Studies investigating pacing behavior in children and junior athletes.

**Study**	**Study design**	**Sport type**	**Exercise type**	**Age group**	**Main findings**
Micklewright et al., 2012	Experimental (cross-sectional)	Run	Time trial (450 m) (600 m) (750 m) (900m)	5.6 ± 0.5 year 8.7 ± 0.5 year 11.8 ± 0.4 year 14.0 ± 0.0 year	Between 9 and 11 years of age, the pacing behavior of schoolchildren develops from a negative pacing behavior, indicating an inability to anticipate exercise demand, toward a more conservative U-shaped pacing behavior, indicating an energy reserve.
Lambrick et al., 2013	Experimental (cross-sectional)	Run	Time trial and head-to-head (800 m)	9–11 year	Unexperienced children presented a *U*-shaped pacing behavior, indicating an energy reserve. Performance was decreased by the introduction of an opponent.
Deaner and Lowen, 2016	Experimental (cross-sectional)	Run	Head-to-head (5,000 m)	High-school athletes	Male runners showed a greater slowing from the first to the second mile of the race, compared to female runners.
Wiersma et al., 2017	Experimental (longitudinal)	Long-track speed skating	Time trial (1,500 m)	U15 U17 U19	The pacing behavior of junior skaters develops, toward a relatively slower start and final round, and a relatively faster mid-section, throughout adolescence. Skaters who reached the elite level as adults exhibit a pronounced development of pacing behavior at a younger age, compared to sub-elites and non-elites.
Menting et al., 2019b	Experimental (cross-sectional)	Short-track speed skating	Head-to-head (1,500 m)	U17 U19 U21 Adults	The pacing behavior of youth skaters changes during adolescence, toward the conservative behavior exhibited by adult skaters. Female skaters tend to adopt the pacing behavior of adult skaters earlier compared to male skaters.
Menting et al., 2019a	Review	Swimming	Head-to-head (200 m)	Junior: 16.9 ± 2.1 year	- Junior swimmers exhibit a more variable pacing profiles compared to adults.
			(400 m)	Adult: 22.8 ± 2.9 year −18.2 ± 2.0 year	- Junior swimmers exhibit difficulty in selecting the most beneficial pacing strategy before a race.

Although the literature on pacing in children and youth is scarce, studies suggest that the pacing behavior of 5–8 year old children, performing an ~4 min running task, is characterized by a decrease in velocity over the duration of the task, pointing to a lack of skill to anticipate the demands of the running task and a difficulty in setting initial exercise pace (Micklewright et al., 2012). At ~10-years of age, observed pacing behavior shifts toward a U-shaped velocity distribution, suggesting that children of this age develop the ability to hold back an energy reserve in order to achieve the set exercise goal (Micklewright et al., 2012; Lambrick et al., 2013). In long-track speed skating (1,500 m), the pacing behavior of elite adult skaters is characterized by a relatively slow start and a fast 700–1,100 m section. At ~15–16 years of age, there is a relatively large shift in the pacing behaviors of junior speed skaters, in which their pacing behavior becomes more closely aligned with the pacing behavior exhibited by adult skaters (Wiersma et al., 2017). Furthermore, junior skaters who exhibit a more conservative pacing behavior, as typically seen in adult skaters, earlier on in their career, will reach a higher performance level during adulthood (Wiersma et al., 2017). Junior short-track speed skaters show a similar development, as their pacing behavior shift during adolescence, to resemble the typically conservative pacing behavior seen in adults, featuring a relatively slow start and fast final laps. Additionally, during adolescence, junior short-track speed skaters learn to adequately integrate environmental cues (e.g., opponents) in their pacing behavior (Menting et al., 2019b). Lastly, a difference in the development of pacing behavior during adolescence was observed between sexes, with female short-track speed skaters displaying the development toward the conservative pacing behavior of adult skaters, earlier in their development process (Menting et al., 2019b). Similarly, female high school athletes exhibited less slowing down between the first and second mile of a 5 km run, compared to their male counterparts (Deaner and Lowen, 2016). Research in elite adults athletes shows that higher performing athletes are characterized with less slowing down during a race (Hanley, 2014), indicating that female high school runners exhibit a more mature pacing behavior compared to males. In swimming, the pacing behavior of swimmers going through adolescence becomes less variable, and swimmers improve their ability to plan their pacing strategy before a race (Menting et al., 2019a). Summarized, the developmental period starting at 10-year of age, and going through adolescence, presents major shifts in pacing behavior in a multitude of sports. The development of pacing behavior is different between the sexes and is (in speed skating) linked to the performance level an athlete reaches as an adult. There are currently no studies investigating the development of pacing behavior in junior athletes in athletic disciplines. However, the occurrence the above described pacing behavior development in a variety of endurance sports would suggest that there is a large chance that the pacing behavior of athletes in athletic disciplines would also develop during adolescence. Therefore, future research is warranted to investigate how the task characteristics of different athletic disciplines influence the above described development of pacing behavior in junior athletes competing in these disciplines.

## Underlying Mechanisms of Pacing Behavior Development in Youth Athletes

The pacing behavior of athletes seems to go through a development during adolescence (Elferink-Gemser and Hettinga, 2017). It is also a period in life when athletes experience their adolescent growth spurt. Girls attain their maximum peak height velocity around 12 years of age, in contrast to boys who are on average about 14 years old (Malina et al., 2004). Adolescence is marked by physiological transformations as well. For example: the left ventricular mass (LVM), which is an important morphological characteristic of the heart (de Simone et al., 1998), is similar in boys and girls until age 9–12 years, but afterwards grows faster in boys, even when expressed relative to body mass (De Simone et al., 1995). Furthermore, the hematological components of oxygen delivery and the oxidative mechanisms of the muscles are also related to body dimensions and muscle mass (Armstrong and Welsman, 2001; Eisenmann et al., 2001), and will therefore be under development during adolescence. The interplay between all these various developing physical attributes influences athletic performance as well as sport-related behavior such as pacing (Elferink-Gemser et al., 2011). The difference in maturation could possibly be the underlying reason for the previously mentioned phenomenon that female athletes adopted the pacing behavior of adult athletes earlier in their development compared to their male counterparts. Maturing athletes will need to adapt their pacing behavior to their developing physical abilities (Elferink-Gemser and Hettinga, 2017). As female athletes mature at a younger age, they could be further along in this adoption process compared to males of the same age. Tracking of maturation status is already part of the talent developmental strategy advised by the IAAF (Dick, 2013). However, the impact of physical maturation on athlete's pacing behavior is still unexplored in literature and could provide opportunities for future research.

Adolescence is not only a time of physical maturation, but also a period of cognitive development. As pacing is essentially a self-regulatory skill, it stands to reason that its development is closely connected to the development of cognitive skills self-regulation of learning (Brick et al., 2016; Elferink-Gemser and Hettinga, 2017). According to prominent authors, self-regulatory skills are comprised of meta-cognitive functions such as the ability to reflect, plan, monitor, and evaluate a goal-directed process as well as aspects of motivation and self-efficacy (Zimmerman, 2006; Jonker et al., 2010). It has been widely accepted that gathering experience of a task positively influences pacing behavior and performance (Foster et al., 2009; Mauger et al., 2009; Micklewright et al., 2010). Elferink-Gemser and Hettinga (2017) suggested that experience gathered during training and competition calibrates and improves the self-regulatory skillset and therefore an athlete's pacing and performance. Moreover, athletes with a higher meta-cognitive skill level were said to make more efficient use of training and competition, extrapolating more learning from a training session, which could then lead to an improvement in pacing behavior and performance (Elferink-Gemser et al., 2016; Jonker et al., 2019). More recently, it has been proposed that core executive functions support the top-down self-regulatory processes involved in pacing by sustaining attention to the planned goal, the inhibition of distractions to the goal and the adaptation of the pacing behavior as a result of external factors (Hofmann et al., 2012; Hyland-Monks et al., 2018). Adding to the rationale of the relation between the self-regulatory skillset and executive functioning in the case of pacing behavior, is the fact that both are closely linked to the pre-frontal cortex (Elferink-Gemser and Hettinga, 2017; Hyland-Monks et al., 2018). The pre-frontal cortex is an area of the brain responsible for planning and decision making, and actively develops throughout adolescence (Casey et al., 2008). Therefore, it seems realistic to assume that the development in pacing behavior witnessed during adolescence could be, at least partially, ascribed to the development of the pre-frontal cortex as well as the increase in exercise experience, influencing the meta-cognitive self-regulatory skills and core executive functions of developing athletes. Following this rationale, developing elite athletes should be encouraged to compete in high level competitions in order to gather experience and calibrate their pacing skillset, emphasizing the importance of the IAAF organized under 18-, under 20- and Youth-championships. In other sports disciplines such as football and speed skating, the monitoring and training of (meta-) cognitive skills is starting to become integrated in talent development and selection programs (Toering et al., 2012). The importance of the relation between (meta-) cognitive skills and the development of pacing behavior and endurance performance presents new opportunities for scientific research, talent development, and coaching in athletics.

## Coaching Pacing Behavior of Youth Athletes: Motivation and the Drive to Perform

Motivation defines athletes' willingness to exert effort during training and competition (Schiphof-Godart and Hettinga, 2017), and needs to be sufficient for enduring a vast amount of training hours (Mallett and Hanrahan, 2004; Gulbin et al., 2010). Research has shown that the motivation of athletes impacts the sensation of fatigue during exercise, therefore influencing how an athlete paces the exercise (Gibson et al., 2003). Moreover, motivation has previously been described as an important component in the self-regulatory learning process (Zimmerman, 2006), and is therefore also expected to be important in pacing behavior development (Elferink-Gemser and Hettinga, 2017). It therefore seems that motivation is a key factor in pacing behavior and should be considered in the framework of pacing behavior development. Although there is currently no literature explicitly studying the impact of motivation on pacing behavior development in youth athletes, lessons can be learned from more general literature regarding motivation, and applied to the coaching of youth athletes.

As pacing is essentially a goal-oriented self-regulatory process, pacing behavior is influenced by the importance of success regarding the goal of the task (Edwards and Polman, 2013; Zimmerman et al., 2017; Wolff et al., 2019). Modifying either an athlete's perception of the importance, probability or controllability of successfully achieving the goal of a task, will thus modify pacing behavior through increased or decreased motivation (Rhoden et al., 2015; Wolff et al., 2019). Factors such as false or deceptive feedback (Williams et al., 2014), encouragements (Edwards et al., 2018), racing an opponent (Konings et al., 2016), modifying the salience of the reward or the probability of attaining a given goal (e.g., to be close to one's personal best, to be close to a top-3 ranking or qualification) (Schiphof-Godart et al., 2018) can enhance performance through by adapting pacing behavior. All of these factors modify either the weight of the “costs” of the task or the “reward” of reaching the set goal, hence increasing or decreasing athletes' motivation to exert effort (Schiphof-Godart et al., 2018). Coaches play an important role in that they have a strong influence in the nature and importance of the goals athletes choose, and can thus increase motivation, and influence pacing behavior, by providing realistic goals that can be largely controlled by the athlete (setting a personal best performance), in contrast to goals that may be partly determined by external factors (such as beating an opponent). Additionally, coaches could provide ways in which athletes will reach the set goal, including: imagining the joy of success (McCormick et al., 2019), positive self-talk focusing on a positive outcome (Gibson and Foster, 2007; Hatzigeorgiadis et al., 2018), but also realistic expectation regarding the fatigue and exhaustion an athlete undoubtedly will experience during a race (Baden et al., 2005), thinking in several if-then scenario's providing athletes with an internal locus of control and feelings of autonomy competence and self-efficacy (Howle et al., 2016). These methods could all lead to increased motivation and potentially assist in optimizing an athletes' pacing behavior. In the long term, sustaining motivation for their sport might be much easier to sustain when athletes like what they do (intrinsic motivation) and feel as if their active participation in sport is an important part of their identity (harmonious passion) (Vallerand et al., 1987; Ryan and Deci, 2000). Athletes who score highly on measures of intrinsic motivation and/or harmonious passion for their sport will be more inclined to spend their energy toward achieving long-term benefits over short term outcomes (such as success) and choose to train in situations that increase their future skills instead of augmenting their immediate chances of winning (Vallerand et al., 1987; Ryan and Deci, 2000). These athletes are likely to base their long term goal-directed energy distribution more on task-oriented goals, aiming at increasing their own performance rather than outperforming others (Pensgaard and Roberts, 2000). This will also affect the athlete's reaction to feedback: task-oriented and intrinsically motivated athletes will welcome feedback and use it to their benefit (Ryan and Deci, 2000; Biddle et al., 2003; Watson et al., 2011). They will see it as an occasion to improve their performance, will react positively (hence increasing the chances of coaches providing feedback) and increase their skills in learning from feedback Factors increasing intrinsic motivation and a harmonious passion for sport are feelings of autonomy, competence and relatedness (Ryan and Deci, 2000; Vallerand et al., 2007). Summarizing, it is advised that in order to optimize pacing behavior development, coaches are advised to monitoring the motivation of their athletes in order to optimize the pacing process, by setting achievable, realistic goals. Additionally, coaches should consider guiding their athletes in developing intrinsic motivation for their sport, by providing them with opportunities to make their own choices, feel competent and be part of a social group with whom they identify and with whom they feel safe and respected.

## Coaching Pacing Behavior of Youth Athletes: Monitoring and Training the Decision Making Process

Monitoring an athlete's pacing behavior, and over a longer time the development of this pacing behavior, has previously been done through three different means. The first is the observation of split times during competitive events. Secondly, during training there is the possibility of monitoring athletes by combining the observation of split times with additional physiological data (e.g., heart rate) and rate of perceived exertion. Lastly, in a controlled laboratory setting, monitoring of power output and the use of different energy systems during exercise can be used to give a detailed description of the pacing behavior of an athlete. Long term monitoring of athletes could, in the future, provide coaches with benchmarks for the development of pacing behavior. In order to monitor the underlying mechanisms related to pacing behavior development, physical maturation (meta-) cognitive development, and the motivation of athletes should be tracked as well. In literature, physical maturation is commonly monitored via the age at peak height velocity, as calculated using sitting height, leg length and body mass (te Wierike et al., 2015). As for the (meta-) cognitive skills associated with pacing behavior development, core executive functions have previously been studied and monitored via a variety of tests, including well known tests such as Tower of London and Stroop task (Jacobson and Matthaeus, 2014). Monitoring of self-regulatory skills in athletes could be done via questionnaires such as the Self-Regulation of Learning Self-Report Scale (Jonker et al., 2010; Toering et al., 2012). As for the monitoring of the motivation of athletes, several questionnaires have been validated and used in research with this purpose, for example: Achievement Goals Questionnaire for Sport (Conroy et al., 2003) or the Behavior Regulation Exercise Questionnaire (Markland and Tobin, 2004).

In addition to monitoring the pacing behavior of athletes, it would be interesting to see how the decision-making processes involved in regulating pacing behavior could be trained. Recent literature suggests that human-environment interactions, such as those experienced when competing against opponents, play an essential role in regulating pacing (Smits et al., 2014; Hettinga et al., 2017; Konings and Hettinga, 2018). The emphasis placed on the human-environment interaction is analogous to contemporary frameworks of sport performance rooted in ecological dynamics (Seifert and Davids, 2017), including the constraints-led approach to skill acquisition (Davids et al., 2008; Renshaw and Chow, 2019). Inherent within these theoretical approaches is the idea that perception and action are causally and cyclically related (Handford et al., 1997; Kugler and Turvey, 2015). In the context of pacing, this concept of perception-action coupling relates to the dynamic process of continuously exploring information from the human-environment interaction to produce the specific affordances (or opportunities for action) that may result in a change of pacing behavior (Smits et al., 2014). Such affordances are predicated by both the perceptual information provided at any given moment in the race (e.g., distance to lead athlete) and the current action capabilities of the individual (e.g., athletes' current physical conditioning/fatigue). Thus, because of the highly dynamic nature of sport, and subsequent human-environment interactions, the development and training of decision making can be viewed as a dynamic and non-linear process (Chow et al., 2013).

From a coaching perspective, the concept of affordances is a central component of the constraints-led approach (CLA) to skill acquisition (Davids et al., 2008). According to proponents of the CLA, it is hypothesized that specific emergent behaviors (e.g., pacing) emerge as a function of three interacting constraints; task (e.g., intensity, loading), individual (e.g., current conditioning, perceived fatigue), and environmental (e.g., heat, altitude) constraints. Through systematically manipulating these constraints, coaches (and athletes) can construct highly representative learning environments to replicate the specific perceptual-motor demands required of athletes to regulate their pace during competition. Due to the centrality of perception-action coupling within the CLA, athletes who have been repeatedly exposed to such highly representative conditions during development (through practice and competition) are thought to develop an adaptable repertoire of perceptual-motor behaviors (e.g., pacing) suited to the dynamic conditions of competition (Pinder et al., 2011; Chow et al., 2013). A practical example can be found in the preparation of athletes for the extreme environmental constraints (e.g., heat, humidity) anticipated at the 2019 IAAF championships in Doha. Of primary concern will be accessing facilities to replicate the specific environmental conditions athletes will face during the championships. Consistent with the CLA, optimal practice conditions will provide athletes with “on-track” race experience against relevant opponents in temperatures like those expected in Doha. For many developmentally elite athletes, directly experiencing these exact conditions is unlikely for many financial and logistical reasons. Consequently, many athletes will be trained in environmental chambers in which the environmental constraints can be externally controlled. While heat acclimatization procedures have been shown to improve physiological performance (Chalmers et al., 2014) the often isolated nature of such training apparatus can result in a decoupling of perception and action. That is, athletes are not exposed to opponents, and are thus less likely to recognize the specific affordances to modify their pacing presented during the dynamic human-environment interactions in extreme environmental conditions. To combat such issues, coaches may utilize innovative methods to increase the fidelity of the practice conditions so that perception and action are more tightly coupled. Recent advances in virtual reality offer exciting opportunities to create immersive and interactive virtual environments, potentially leading to greater decision making transfer to competition through perception action coupling (Craig and Cummins, 2015; Stone et al., 2019). Less expensive methods, such as projecting different competitive scenario's using life size images of opponents using video or avatars on to screens, from the athlete's perspective, may also be advantageous in enhancing perception-action coupling (Savelsbergh et al., 2002, 2010; Croft et al., 2013; Konings and Hettinga, 2018). Despite the potential of such novel training methods, the unique psycho-social environmental constraints experienced by developing athletes attending their first senior world championships is virtually impossible to recreate. Yet, the experience will be invaluable in recognizing the specific affordances to regulate their pacing behavior in future IAAF World Championship events.

## Conclusion

Pacing vitally impacts athletic performance both during competition and over a longer period of time. It is therefore imperative that young athletes, striving to reach the elite level, adequately develop their pacing behavior. Adolescence is characterized by major shifts in pacing behavior. Pacing can be seen as a self-regulatory skill and the development of pacing behavior during adolescence is thought to be underpinned by physical maturation, the development of pre-frontal cortical (meta-) cognitive functions, as well as the increase of experience with exercise tasks. Motivation impacts self-regulatory learning and is of importance for optimal development of pacing skills. Coaches are advised to monitor the motivation of young athletes and encourage a motivational climate in which realistic goals are set, athletes enjoy their sport, create positive relationships (with coach, staff, and teammates) and value long term decisions over short term success. Monitoring and training the development of pacing behavior, by ways of a constrained led approach, could be the next step in the talent development process. The complexity of pacing behavior development, as well as its essential role in the career of elite athletes, warrants the need for further research. Future studies are needed to establish benchmarks for pacing behavior development of elite athletes in specific athletic disciplines, as well as a further exploration of the underlying mechanisms of the development of the pacing skillset in the elite athletes of tomorrow.

## Author Contributions

SM wrote the first draft of the manuscript. DH, LS-G, ME-G, FH, and SM wrote sections of the manuscript. All authors contributed to conception and design of the work, drafted it, and revised it critically for important intellectual content. All authors contributed to manuscript revision, read and approved the submitted version. All authors have approved the final version of the manuscript, agree to be accountable for all aspects of the work in ensuring that questions related to the accuracy or integrity of any part of the work are appropriately investigated and resolved. All persons designated as authors qualify for authorship, and all those who qualify for authorship are listed.

### Conflict of Interest Statement

The authors declare that the research was conducted in the absence of any commercial or financial relationships that could be construed as a potential conflict of interest.
